# A Novel Deep-Learning Method with Channel Attention Mechanism for Underwater Target Recognition

**DOI:** 10.3390/s22155492

**Published:** 2022-07-23

**Authors:** Lingzhi Xue, Xiangyang Zeng, Anqi Jin

**Affiliations:** School of Marine Science and Technology, Northwestern Polytechnical University, Xi’an 710072, China; 2018100384@mail.nwpu.edu.cn (L.X.); jinaq@mail.nwpu.edu.cn (A.J.)

**Keywords:** feature extraction, target recognition, neural networks, underwater acoustic signals

## Abstract

The core of underwater acoustic recognition is to extract the spectral features of targets. The running speed and track of the targets usually result in a Doppler shift, which poses significant challenges for recognizing targets with different Doppler frequencies. This paper proposes deep learning with a channel attention mechanism approach for underwater acoustic recognition. It is based on three crucial designs. Feature structures can obtain high-dimensional underwater acoustic data. The feature extraction model is the most important. First, we develop a ResNet to extract the deep abstraction spectral features of the targets. Then, the channel attention mechanism is introduced in the camResNet to enhance the energy of stable spectral features of residual convolution. This is conducive to subtly represent the inherent characteristics of the targets. Moreover, a feature classification approach based on one-dimensional convolution is applied to recognize targets. We evaluate our approach on challenging data containing four kinds of underwater acoustic targets with different working conditions. Our experiments show that the proposed approach achieves the best recognition accuracy (98.2%) compared with the other approaches. Moreover, the proposed approach is better than the ResNet with a widely used channel attention mechanism for data with different working conditions.

## 1. Introduction

The traditional methods of target recognition include feature extraction techniques based on mathematical modeling [1]. Using the entropy theory [2,3] as a feature to extract the radiation noise of a ship is one of the most common mathematical modeling methods. Additionally, a critical approach to recognition is to analyze the peaks of the spectrum to obtain the physical features, such as the propeller speed cavitation noise of the engine [4,5]. The spectrum will be distorted because of the Doppler effect when the ship moves toward the hydrophone receivers [6]. Wang proposes the multi-method spectra based on auditory feature extraction from the human ear and effectively extracts stable feature points under the Doppler effect [7]. Modeling the Doppler power spectrum of non-stationary underwater acoustic channels is another method to reduce the impact of the Doppler effect in underwater acoustic target recognition [8]. The information extracted by traditional methods is limited when the spectrum of signal changes with the Doppler effect. Li [9] uses the square root unscented Kalman filter to attenuate the Doppler phenomena in underwater acoustic signals.

Deep learning has an advantage in extracting the spectrum feature compared with the traditional method. However, it is often difficult to collect enough underwater acoustic signal data for training, which significantly limits the performance of deep neural networks in underwater target recognition. Nevertheless, researchers are still exploring the application of deep learning in underwater target recognition with the constraints of the available underwater acoustic data. Yang [10] et al. use deep auto-encoder networks combined with long short-term networks to extract target features and set different gates according to the temporal characteristics of underwater acoustic to extract feature information effectively. Auto-encoder networks can downscale high-dimensional data to low-dimensional data while retaining sufficient feature information, but the number of parameters is enormous. The convolution neural network (CNN) method can significantly reduce the number of parameters compared with DNN. Hence, CNN is better for underwater acoustic signals with limited samples. Hu [11] uses CNN to reduce the number of parameters and obtain better experimental results. Wang [12] investigates the intrinsic mechanism of convolution networks for underwater acoustic signals and displays the relationship between the waveform of the original data and the convolution kernel. Hu builds an underwater acoustic recognition model based on separable convolution operations according to the information collection mechanism of the auditory system, which is the first time grouped convolution models are applied in underwater acoustic recognition. Tian [13] applies a deep convolution stack to optimize CNN networks, which solves the lack of depth and structural imbalance of the networks. However, the above CNN model extracts single-scale features with the fixed size of the convolution kernel, which lose a lot of feature information. Hong [14] proposes a deep convolution stack network with a multi-scale residual unit (MSRU) to extract multi-scale features while exploring using generative adversarial networks (GAN) to synthesize underwater acoustic waveforms. The method modifies two advanced GAN models and improves their performance. GAN network with generators model and adversaries model uses the idea of the game to optimize the network. The generators can generate underwater acoustic samples when reaching the Nash equilibrium between the generators and adversaries models. We propose [15] an underwater acoustic target recognition model based on GAN, optimizing the recognition model with two model adversaries. The experiment verifies the better recognition ability of GAN than the other networks with small samples. The number of neural network layers has increased due to the urgent need to identify underwater acoustic data under different spatial and temporal conditions. Doan [16] applies the dense convolutional neural network to identify the target class, which addresses the over-fitting problem in a deep convolutional neural network with a limited number of samples. Gao [17] increases the number of samples using the GAN model, extracting underwater acoustic features with small samples in deeper network layers. To solve the recognition problem with a limited number of samples in deep networks, He [18,19] first proposes a ResNet model in image recognition, which uses the residual function to eliminate the gradient disappearance effectively. Wu [20] conducts deeper research in terms of the depth and width of ResNet models. Liu [21] applies the ResNet model to the study of underwater acoustic signals and acquires good experimental results.

Hu [22] first proposes the SE (squeeze and excitation) network, which uses channel weighting to discriminate the importance of information in different channels of ResNet. This network is a channel attention mechanism approach that can assign weights to different channel information according to their effectiveness and effectively remove channels with similar features. The channel attention mechanism can again adaptively optimize the neural network models, and different channel attention mechanisms for different research objects are required. Because of the low-pass filtering properties of the underwater acoustic channel, the high-frequency spectrum of the signal is decreased when increasing the distance between the target signal and the hydrophone. So, the underwater acoustic signal contains the spectrum of low frequency and the continuous spectrum. The continuous spectrum contains the ocean background noise signal, and the spectrum of low frequency contains the ship’s radiation noise, propeller noise, machine noise, and other hull self-noise. Yang [23,24] uses an auditory inspired for ship type classification. The core of the underwater acoustic target recognition method is to extract the low-frequency spectrum [25,26]. However, the distance changes between the target and the hydrophone lead to a Doppler shift, which makes the information in the low-frequency spectrum disappear. This paper designs a camResNet (ResNet with channel attention mechanism) model to extract the low-frequency spectrum of underwater acoustic signals when the Doppler shift occurs. The channel attention mechanism of camResNet is divided into two parts. First, the signal channels are weighted by analysis of channel information. Second, the valid information points in each channel are extracted, and the complete information is weighted.

This paper is organized as follows. Section 2 introduces the structure of the SE_ResNet network. Section 3 describes the details of the underwater acoustic target recognition method based on camResNet. Section 4 describes the experimental data and shows the experimental results. Section 5 concludes the advantages and disadvantages of the proposed method.

## 2. Structure of ResNet

The ResNet model deals with network degradation caused by network layer deepening using residual learning methods. Hong [27] studied the characteristics of underwater acoustic signals and increased the recognition rate with an 18-layer residual network (ResNet18), which contains an embedding layer.

**Figure 1:**
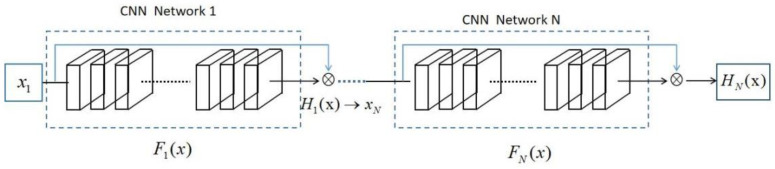
The architecture of the ResNet model.

The ResNet model consists of many residual modules; the input of the modules is *x*, and the output of the convolutional structure of multi-layer stacking is H(x), called the learned features. The learned features are difficult to optimize by backward gradient propagation with a network having too many layers, even if the nonlinear activation function performs very well. He finds that function F(x)=H(x)−x, called the residual function, is easier to optimize H(x). The output of residual modules is the complex feature function F(x)+x, which is the residual function learned by the network summed with the original signal, and the output of residual modules is the input of the following residual modules. Figure 1 shows the architecture of the ResNet model, in which H(x) is the residual function, and the mathematical expression is defined as

(1) $$H\left(x\right)=x+w_{N}\delta \left(w_{N-1}\left(\delta \left(\ldots \delta \left(w_{1}x\right)\right)\right)\right)$$

The $w_{1}\cdots w_{N}$ in this equation denotes the weight of each module in the residual network. The function for x mathematical expression is defined as

(2) $$\frac{\partial H\left(x\right)}{\partial x}=1+\frac{\partial \left(w_{N}\delta \left(w_{N-1}\left(\delta \left(\cdots \delta \left(w_{1}x\right)\right)\right)\right)\right)}{\partial x}$$

The first term of Equation (2) equals 1, and the second term is the gradient value of the weight function to x. Since it contains 1, the function $\frac{\partial H\left(x\right)}{\partial x}$ will not equal 0, even if the second term is small.

## 3. Architecture of camResNet in Underwater Acoustic Target Recognition Method

### 3.1. Architecture of camResNet

**Figure 2:**
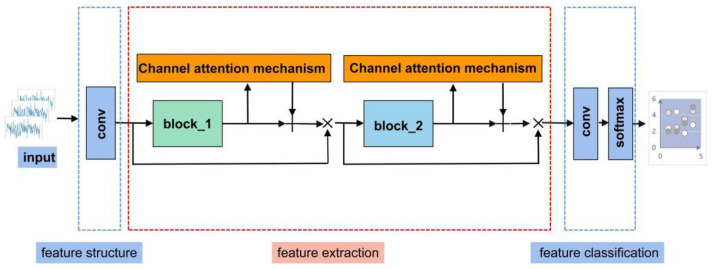
The architecture of the camResNet model.

The camResNet model is excellent for extracting classification-related feature information because it adds the channel attention mechanism based on the ResNet model. The process of the camResNet model includes three steps: feature structure building, feature extraction, and feature classification, as shown in Figure 2.

The low-dimensional underwater acoustic signal limits the ability of convolution networks to extract high-dimensional abstract features. So, the feature structure building module decomposes the input acoustic signal into base signals using a set of one-dimensional convolutions as deep convolution filters, which can obtain high-dimensional input data. Different convolution kernels of N are set in the deep convolution filters $F\left(F_{1},F_{2}\cdots F_{N}\right)$, and each convolution layer contains a two-dimensional convolution kernel. The output of the feature module contains 16 groups of signals, so 16 one-dimensional convolution layers are needed. The specific formula is as follows:

(3) $$y_{i}^{m}=f\left(x^{m}\times \omega_{i}^{m}+b_{i}^{m}\right)$$

where $x^{m}$ is the *m*-th input sample, $\omega_{i}^{m}$ denotes the convolution kernel of the *i*-th output channel of the *m*-th sample, $b_{i}^{m}$ denotes the bias function of the *i*-th output channel of the *m*-th sample, and $y_{i}^{m}$ is the *i*-th channel output value of the *m*-th sample. The symbol × means dot product. Finally, the output feature group of the *i*-th layer is $y_{i}^{m}$, formed through the ReLU function f(⋅).

The number and frequency of the spectrum are the primary basis for underwater acoustic signal target recognition. The spectrum energy that will shift with the change of distance between the target and the hydrophone is called unstable spectra. The spectrum energy that will not shift with the change of distance between the target and the hydrophone is called stable spectra. The camResNet model can extract the stable spectrum of the underwater acoustic target as the feature to recognize the target category accurately when the spectra of the target are shifted due to the Doppler effect. The stable spectra contain many harmonic signals. The fundamental frequency is the shaft frequency signal of the propeller, and the relationship of the harmonic groups is the multiplier. For a B-bladed propeller, each B is a set of pulses with a period *T*, and the repetition period of the pulses is *T*/B. The 2*N* + 1st set of pulses in the time domain signal is selected, and its *k*-th Fourier transform is denoted as $F_{N}^{k}\left(\omega \right)$. The specific formula of power spectral density by this random process is as follows [28]:

(4) $$\begin{array}{ll} & s\left(\omega \right)=E\left\{s_{k}\left(\omega \right)\right\}=E\left\{\underset{N\to \infty }{\lim}\frac{1}{\left(2N+1\right)T}{\left|F_{N}^{\left(k\right)}\left(\omega \right)\right|}^{2}\right\}\;\\ & ={\left|g\left(\omega \right)\right|}^{2}\left\{\left(2N+1\right)\left(U_{1}-U_{2}\right)+\overset{2N}{\underset{p=-2N}{\sum }}\left(2N+1-\left|p\right|\right)\cos\omega pT[U_{2}+U_{3}\cdot 2\cos\omega \left(T/2\right)+U_{4}\cdot \cos\omega \left(T/4\right)]\right\} \end{array}$$

where E{⋅} is the expected value, ω denotes angular frequency g(ω) Fourier spectrum, representing the time domain waveform. The specific formula of *U* is as follows:

(5) $$\{\begin{array}{l} U_{1}=\bar{a_{0}^{2}}+\bar{a_{1}^{2}}+\bar{a_{2}^{2}}+\bar{a_{3}^{2}}\\ U_{2}={\bar{a}}_{0}^{2}+{\bar{a}}_{1}^{2}+{\bar{a}}_{2}^{2}+{\bar{a}}_{3}^{2}\\ U_{3}={\bar{a}}_{0}\cdot {\bar{a}}_{1}+{\bar{a}}_{1}\cdot {\bar{a}}_{3}\\ U_{4}={\bar{a}}_{0}\cdot {\bar{a}}_{1}+{\bar{a}}_{1}\cdot {\bar{a}}_{2}+{\bar{a}}_{2}\cdot {\bar{a}}_{3}+{\bar{a}}_{3}\cdot {\bar{a}}_{0} \end{array}$$

where $a_{i}$ denotes the amplitude of the pulse number i in a set of signals. ${\bar{a}}_{i}$ denotes the average value of $a_{i}$. The fundamental frequency and the first group of harmonic signals can be used as stable signal characteristics because the modulation spectrum of the actual vessel radiation noise decays rapidly with the increasing number of groups of spectra. The obtained multidimensional information with the feature structure building module is called the original information, which is the input of the feature extraction module. The feature extraction module contains two ResNet models with the channel attention mechanism. A convolution kernel size of 1×64 is a good trade-off between the quality of the recognition and the computational cost of the model for underwater acoustic. The first layer of the residual network contains two convolutions. Each convolution operation maps 16 sets of base signals to another 16 sets of base signals to extract the deep features of the signal. The convolution operation consists of 16 convolution layers, each containing 16 different filters $F\left(F_{1},F_{2}\cdots F_{N}\right)$. So, 16×16 one-dimensional convolution layers are needed. The specific formula is as follows:

(6) $$y_{i}^{m}=\overset{N}{\underset{k=1}{\sum }}f\left(x_{k}^{m}\times \omega_{ik}^{m}+b_{ik}^{m}\right)$$

where $x_{ik}^{m}$ denotes the input value of the *k*-th channel in the *m*-th sample, $\omega_{ik}^{m}$ denotes the *k*-th convolution kernel of the *i*-th layer convolution of the *m*-th sample, $b_{ik}^{m}$ denotes the *k*-th bias function of the *i*-th layer convolution of the *m*-th sample, and $y_{ik}^{m}$ is the output of the *i*-th layer convolution of the *m*-th sample. The symbol × means dot product. The output feature group of the *k*-th convolution of the *i*-th convolution layer is formed through the activation function f(⋅), which uses the ReLU function.

Finally, all the convolution outputs in the *i*-th layer are summed up as the convolution output value of the *i*-th layer. The second convolution is the same as the first convolution operation in order to obtain deeper underwater acoustic features. A channel attention mechanism is added to each one-residual network to enhance the stable spectrum features and further enhance the network’s performance in extracting underwater acoustic signals. 

Section 3.2 describes the channel attention mechanism of the feature structure building module in detail.

The feature classification uses a fully convolutional network to map the high-dimensional features from the output of the feature extraction module to a lower dimension with the size of the classification class. The details are listed as follows.

Stage 1: In feature structure, the data shape of the input layer is a four-dimensional matrix 64×1×1×800. The shape changes from 64×16×1×800 to 64×16×1×800 by convolutional layer. The batch normalization layer is applied, followed by a ReLU activation function and max pooling with the stride of 2 × 1.

Stage 2: The feature extraction module contains two residual modules, called block-1 and block-2. The input shape of block-1 is 64×1×1×400. The shape changes from 64×16×1×400 to 64×16×1×400 by two convolutions with a convolution kernel of 64×1 and a stride of 1×1. Batch normalization is applied after each convolution and connected between the two convolutions using the activation function ReLU. Finally, add the channel attention mechanism, marked with the dashed yellow box in Figure 2, which will be described in detail in Section 3.2 of the paper. The obtained data are summed with the original data as the output of block-1.

Stage 3: The input shape of block-2 is 64×1×1×400. The shape changes from 64×16×1×400 to 64×16×1×200 by convolution with a convolution kernel 64×1 and a step of 2×1. Batch normalization and a ReLU activation function are applied. The second convolution does not change the shape of the data and adds the channel attention mechanism. The obtained data are summed with the original data as the output of block-2.

Stage 4: This paper uses a fully convolutional networks model, in which a cubic convolutional network is used to map high-dimensional features to low-dimensional features in the decision module.

### 3.2. Structure of Channel Attention Mechanism Based on Underwater Acoustic of camResNet

The changes in the distance between the target and the hydrophone lead to a Doppler effect, which is the frequency move. The Doppler frequency compensation is challenging, as the underwater acoustic channel is low-frequency filtering. The method in this paper can extract the stable spectral features under the Doppler frequency shift by the channel attention mechanism, which can automatically acquire the critical information in each feature channel by learning to enhance the valuable features and suppress the less useful features for the current task. 

**Figure 3:**
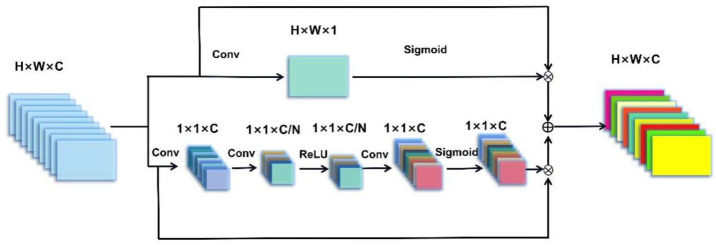
Channel attention mechanism network model.

The amount of information on the channels is different, and the channel attention mechanism increases the weight to that of the channel with high information. It can improve the model’s capability. First, squeeze the information out of each channel and then add a lightweight gating system to optimize the channel information and output the channel weights. The channel attention mechanism of this paper is divided into two parts. Figure 3 shows the channel attention mechanism model. The first part is the primary part, which weighs each channel, and the second part is the auxiliary part of formation extraction, which is another channel information after transposing the information.

The first part analyzes the waveform features in each channel separately. First, process the data with a convolution kernel H×W and the stride of W; the shape changes from H×W×C to 1×1×C. Where H represents the length of the input data, W represents the width of the input data. The specific formula is as follows:

(7) $$x^{\left(m\right)}=\overset{M}{\underset{i=1}{\sum }}\overset{N}{\underset{k=1}{\sum }}f\left(x_{k}^{\left(m\right)}\times \omega_{ik}^{\left(m\right)}+b_{ik}^{\left(m\right)}\right)$$

where $x_{k}^{\left(m\right)}$ denotes the *k*-th channel data in the input information of the channel attention mechanism module, $\omega_{ik}^{\left(m\right)}$ denotes the weight of the *k*-th channel of the *i*-th layer of convolution, $b_{ik}^{\left(m\right)}$ denotes the bias of the *k*-th channel of the *i*-th layer of convolution, and $x^{\left(m+1\right)}$ denotes the output value of $x^{\left(m\right)}$ after one convolution.

The data of each channel characterize the global features of each channel. In order to be able to learn the nonlinear characteristics between the channels independently, this paper uses a gating system with an activation function. The specific formula is as follows.

(8) $$x^{\left(m+3\right)}=\overset{M}{\underset{i=1}{\sum }}\overset{N}{\underset{k=1}{\sum }}\sigma \left(\delta \left(x_{k}^{\left(m+1\right)}\times \omega_{ik}^{\left(m+1\right)}+b_{ik}^{\left(m+1\right)}\right)\times \omega_{ik}^{\left(m+2\right)}+b_{ik}^{m+2}\right)$$

where $x_{k}^{\left(m+1\right)}$ is the global feature of size 1×1×C. $\omega_{ik}^{\left(m+1\right)}$ and $\omega_{ik}^{\left(m+2\right)}$ are the weights of the network mapping. In order to obtain the features of the network channel, convolutional mapping is used, and the feature points before mapping are *r* times after mapping, so $\omega_{ik}^{\left(m+1\right)}\in R^{\frac{c}{r}\times c}$, $\omega_{ik}^{\left(m+2\right)}\in R^{c\times \frac{c}{r}}$. δ is the ReLU activation function, and σ is the sigmoid activation function.

The second part synthesizes the signal characteristics in all channels. Process the data with a convolution kernel 1×64 and the stride of 1; the shape changes from H×W×C to H×W×1. The multi-layer convolutional network has a solid ability to extract sufficient recognition information, and the output of the network contains a large number of stable signals with a small number of unstable signals. One-dimensional data of the same size are extracted from the network’s output as the channel weights of the original signal, which can effectively enhance the spectrum energy contained in the channel.

The two parts of the channel attention mechanism weigh the signal features from different perspectives. Finally, the two weighted pieces of information are fused as the output of the channel attention mechanism. 

## 4. Model Evaluation

### 4.1. Dataset

The eight hydrophones are fixed at the same level in eight different places at the same interval. This paper randomly selects four sets of hydrophones at equal intervals as input data. The data used in the experiments contain four classes of vessels, and the third of the four types of signals is the radiated noise of the iron vessel, while the first, second and fourth types are vessels of the same material and similar hull size. 

**Figure 4:**
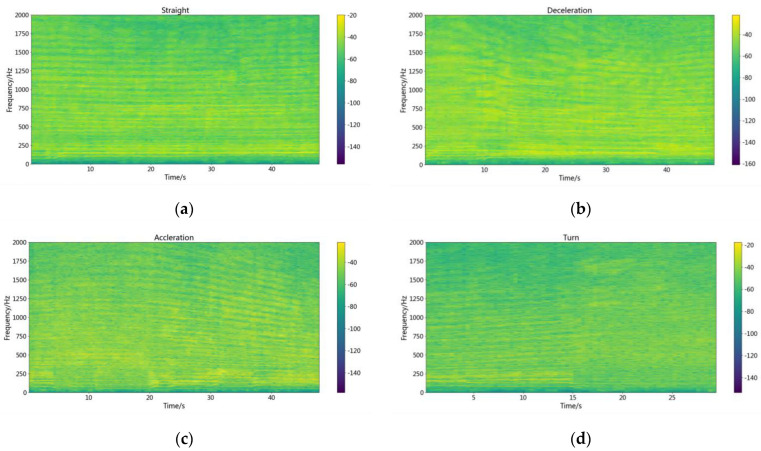
The spectrogram by the fourth type of vessel. (**a**) the spectrogram of straight motion. (**b**) the spectrogram of decelerating motion. (**c**) the spectrogram of accelerating motion. (**d**) the spectrogram of turning movement.

To study the recognition effect of camResNett under different Doppler frequency shifts, four different working conditions were intercepted in each class of experimental data. Each class of the data obtained has four modes of operation: straight ahead at a constant speed, straight forward acceleration, straight-ahead deceleration, and turning. Figure 4 shows the spectrogram of different working conditions by the fourth type of vessel.

Figure 4a is the time-frequency relationship of the signal by the vessel of straight motion. It shows that there is acceleration when the vessel is just starting, and the frequency shifts to high frequency. The speed reaches stability within a brief period, and a stable spectrum characteristic appears, which contains line and continuous spectra. The formula with the Doppler shift is as follows:

(9) $$f=f_{0}\cdot \frac{1}{1-\frac{u\cos\theta }{v}}$$

where $f_{0}$ is the original frequency of the vessel, v is the speed of the underwater acoustic signal propagating in the channel, *u* is the speed of the vessel motion, and *f* is the frequency after the Doppler shift. θ is the angle between the line of the vertical distance connecting the ship and the hydrophone and the line connecting the ship and the hydrophone. The signal will have a stable frequency shift when the vessel movement speed is constant. In the passive recognition process, the stable spectrum feature after the frequency shift is the primary information for recognizing the target. However, when the target accelerates, the u keeps changing, and the f varies with the change of u. Figure 4b,c are time-frequency diagrams of the ship in the motion state of acceleration and deceleration. The low-frequency spectra are the stable spectra, and the spectrum above 400 Hz will change with time. Figure 4d is the time-frequency diagram by the vessel of turning, and a large number of unstable spectra appear in the time-frequency diagram because the θ keeps changing.

**Figure 5:**
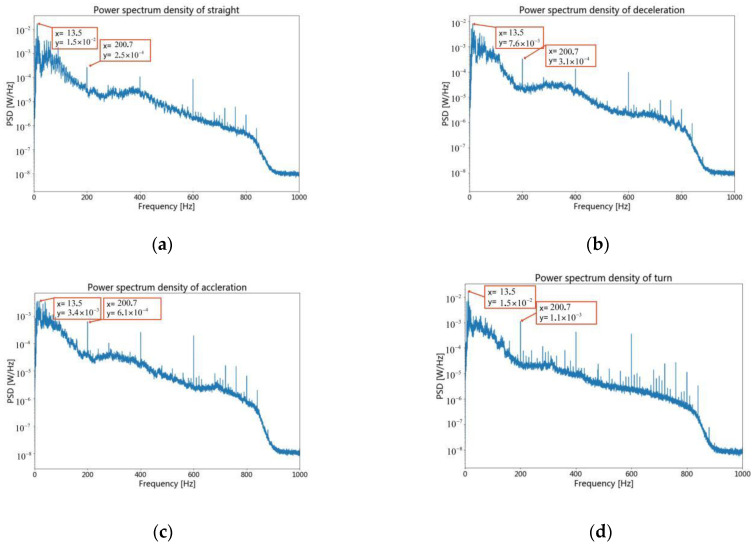
The power spectrum density by the fourth type of vessel. (**a**) The power spectrum density of straight motion. (**b**) The power spectrum density of decelerating motion. (**c**) The power spectrum density of accelerating motion. (**d**) The power spectrum density of turning movement.

To further observe the energy distribution of the frequencies with the vessel for different operating conditions, Figure 5 shows the power spectral density for the different operating conditions by the fourth type of vessel, which is the Fourier transform of the correlation function with the 0.5 s window length.

Figure 5 shows the power spectrum density by the fourth type of vessel. A set of resonant waves at a fundamental frequency of 200 Hz occur stably under four different operating conditions. High-frequency points are shifted when the vessel is in an accelerated motion. The high-frequency spectral density varies significantly, and the low-frequency spectral density is more stable than the high frequency under different working conditions. Figure 5b,c show the acceleration and deceleration. Compared with Figure 5a, the power spectral density in high frequency is higher than in the straight motion, and some frequency points in the high frequency are changed. Figure 5d shows turning, and many spectral density power spikes appear in the high frequency compared with Figure 5a.

The same class of targets contains different Doppler shift signals, which will increase the difficulty of recognition, with the original signal compressed or broadened. This method extracts the stable features of the same class of vessels under different working conditions. 

**Figure 6:**
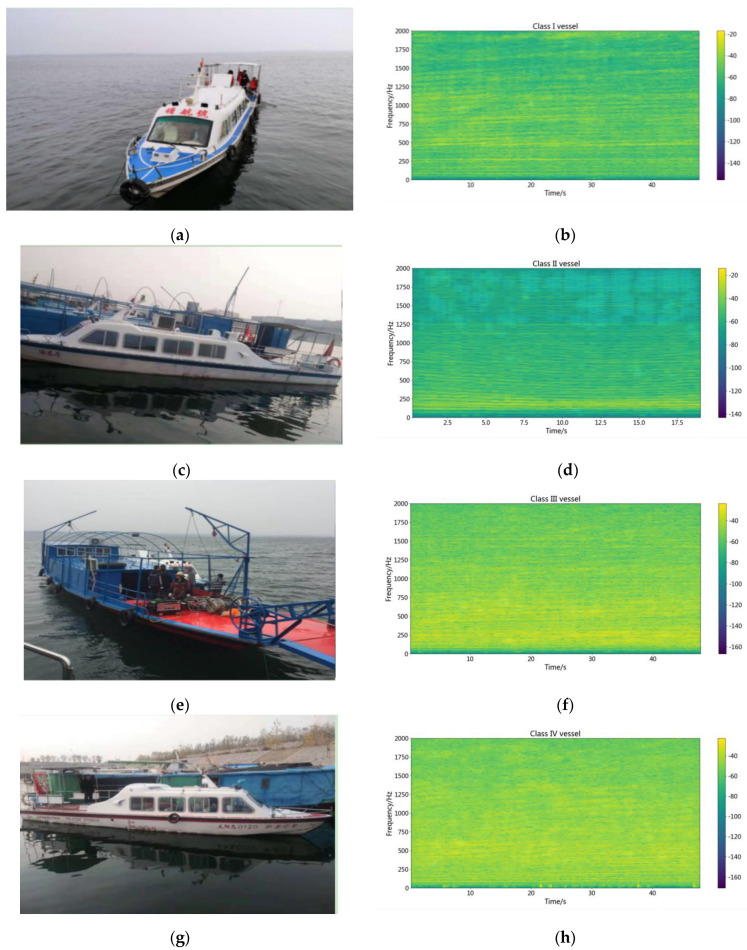
The pictures and spectrograms of the different vessels. (**a**) the pictures of class I vessel. (**b**) the spectrogram of class I vessel. (**c**) the pictures of class II vessel. (**d**) the spectrogram of class II vessel. (**e**) the pictures of class III vessel. (**f**) the spectrogram of class III vessel. (**g**) the pictures of class IV vessel. (**h**) the spectrogram of class IV vessel.

To study the difference between the categories with four types, the straight motion working condition of each type of vessel is chosen to exhibit a time-frequency relationship. Figure 6 shows the pictures and time-frequency diagrams of the four types of vessels, containing class I, class II, and class III and IV vessels. The background noise of the four vessels has relatively apparent differences, but there are similar low-frequency spectra.

As can be observed in Figure 6b,d, a clear line spectrum in the low-frequency band is very similar. Figure 6h has two precise line spectra, respectively, similar to the line spectra in Figure 6b,d. No clear line spectrum is observed in Figure 6f, but the energy distribution at low frequencies is similar to that in Figure 6h. Figure 6 shows that the spectrum is very similar to the different vessel types, in which the spectrum energy is concentrated in the low frequency and continuous. So, it is difficult to distinguish the vessel category with the traditional method.

### 4.2. Data Pre-Processing

There are 800 feature points (0.1 s) for a frame and no overlap between frames. If the maximum feature point of the sample is less than 0.1, eliminate the small value frame sample, ensuring that the recognition results are not affected by the particular sample points. After eliminating the small samples, the samples contain 7097 samples. Use 1/4 of the data as the test set and 3/4 of the data as the training set after normalizing the samples. The prepared data have 9462 samples as the training set and 1774 samples as the test set. In total, 200 samples are randomly selected as the validation set in each class, and the validation set contains 800 samples in total. The training method is a batch method, in which 64 samples are randomly selected in each batch, and the selected samples will not be used as alternative samples in the next batch. 

### 4.3. Experimental Results

#### 4.3.1. Discussion of Model Structure

This reports the experimental results of the model with Doppler shifts signals. The straight condition is considered a signal without a Doppler shift. The other conditions are considered a Doppler shift. The experiment chose four conditions as input data.

The first experiment illustrates the relationship between the recognition rate and the number of residual layers, where the size of the number of residual layers changes in the set of {1,2,3,4}. According to the results in Table 1, two residual layers have the best recognition effect, and the recognition rate will decrease by increasing the number of residual layers. 

Two residual layers are appropriate for the number of samples in the experiment, and the different number of samples matches the different number of layers. If the ResNet network is not over-fitted or under-fitted, the over-fitting phenomenon will occur and decrease the recognition accuracy when adding the channel attention mechanism. If the ResNet network is under-fitted, adding the channel attention mechanism will compensate for this under-fitting phenomenon. The number of model parameters needs to match the number of samples, and the number of parameters increases after adding the channel attention mechanism.

The second experiment illustrates the relationship between the recognition rate and the size of the convolutional kernel. The size of the 1D convolutional kernel varies in the set of {3, 5, 7, 9, 11, 15, 17, 21, 25, 33, 41, 49, 57, 64, 75, 85, 95}. Table 2 shows that a kernel size of 64 is best for the recognition rate. The scale size of the target needs to match the actual perceptual field after the addition of convolutional kernels because the underwater acoustic target is submerged in background noise, and a large amount of ocean background noise is extracted if there is no match.

#### 4.3.2. Classification Experiment Results

In the experimental data, four-vessel classes are used to train different deep-learning network models, and the information of each network model is described below.

(1) The DBN model has an input layer, three hidden layers, and one output layer. The number of nodes in the input layer is 199, the number of nodes in the three hidden layers is 100, 50, and 20, and the number of nodes in the output layer is the number of sample categories. Each pair of adjacent layers constitutes an RBM network, and the three RBM networks are trained separately first, followed by the whole network. A batch method with a batch size of 64 is used for training. A gradient descent algorithm with a learning rate of 0.01 is used to optimize the training process.
(2) The GAN network model consists of two modules: generation and discrimination. The generation module consists of three convolutional layers, and the discrimination module consists of convolutional layers. The generative module comprises three convolutional layers, with 64, 128, and 800 filters with a filter size of 1×4 and a step size of 4. The discriminative model is a single-layer convolutional neural network with 16 filters with a filter size of 1×4 and a step size of 4. Batch training with a batch size of 64 is used, and the learning rate is 0.001.
(3) The DenseNet model is made up of three modules, each of which has three layers of a convolutional neural network. The data are normalized before each convolutional operation, and after convolution, the data are nonlinearly mapped using the elu activation function. The convolutional operation with a convolutional kernel size of 1×64 and a step size of 1 is chosen. The batch method with a batch size of 64 is used for training. The optimization method is chosen during training using a gradient descent method, and the learning rate is 0.001. For optimization, the gradient descent algorithm is used.
(4) The U_Net model is made up of three down-sampling modules and three up-sampling modules. Each down-sampling module contains two convolutional layers and a pooling layer of the specified size of 1×2. There is a splicing layer, a deconvolution layer, and a pooling layer with a pooling size of 1×1 in each up-sampling module. The batch method is used for training, with a batch size of 64 and an optimization method of gradient descent with a learning rate of 0.001.
(5) The SE ResNet network is set up and trained in the same way as the camResNet UAS model network, with the exception that the channel attention mechanism is a three-layer auto-encoder network model.

A test set was used to evaluate the model’s recognition ability. Table 3 shows the recognition rate with straight motion and four different working conditions. The recognition rate of amRestNet and SE_ResNet are similar when the data contain straight data. The recognition rate of amRestNet is higher than SE_ResNet when the data contain four different working conditions. Both amRestNet and SE_ResNet can extract valid feature information when the data contain a single working condition. However, the SE_ResNet is not as effective as amRestNet in extracting stabilization features when different working conditions are included and have different Doppler frequencies.

Table 3 shows that the camResNet model has a recognition rate of 98.2%, which is 1.1–15.8% higher than the other networks. The DBN model is a basic neural network model based on probabilistic statistics, and its input signal is a frequency domain signal. The GAN model is the adversarial model, which mainly contends with small-sample data, and its input signal is the time domain signal. The DenseNet model can simplify the network complexity and reduce network parameters by designing the dense block, and its input signal is the frequency domain signal. The ResNet model uses residual learning to update the network parameters, and its input signal is the time domain signal. The U_Net model uses up-sampling and down-sampling to extract multi-scale features, which can improve the recognition effect, and its input signal is the time domain signal.

The DBN model has different optimization methods compared to other models, which use probabilistic models to optimize the parameters, so the recognition rate of the DBN model is lower than other networks. The recognition rate of U_Net is lower than the GAN model and the DenseNet model because the up-sample and down-sample can lose some feature information. The SE_ResNet model has an excellent performance in recognition rate because the ResNet model has the balance between network depth and recognition rate of small samples. The camResNett model is better than the other models in terms of the recognition rate because the channel attention mechanism deals with underwater signals’ sparsity and multi-scale characteristics.

In the display of recognition experiment results, we use recognition accuracy, recall rate, precision, and F1-score to evaluate the recognition performance of the networks. The formulae for each indicator are as follows.

(10) $$\mathrm{Pr}ecision=\frac{TP}{TP+FP}$$

(11) Recall=TPTP+FN

(12) $$Accuracy=\frac{TP+TN}{TP+TN+FP+FN}$$

(13) $$F1-score=\frac{2TP}{2TP+FP+FN}$$

*TP*, *TN*, *FP*, and *FN* are true positive, true negative, false positive, and false negative. Table 4 shows the precision, recall rate, F1-score, and accuracy of the test sample, while Table 5 shows the confusion matrix.

Class I of the vessel includes three acceleration signals, three deceleration signals, five straight-ahead signals, and seven turn signals. Class II of the vessel includes three acceleration signals, three deceleration signals, three straight-ahead signals, and six turn signals. Class III consists of three acceleration signals, deceleration signals, straight-ahead signals, and five turn signals. Class IV consists of three acceleration signals, deceleration signals, straight-ahead signals, and turn signals. The vessels of the different categories have similar sizes but different materials, and the third category material is significantly different to the materials from the other three. In Table 5, the probability of incorrectly recognizing Class II of the vessel as Class III of the vessel is the highest. This is followed by the probability of incorrectly recognizing Class III of the vessel as Class II of the vessel. This indicates that camResNet extracts shallow physical features and deep category features, which is related to the Doppler effect. Class II of the vessel and Class III of the vessel contain the most similar samples in the composition structure of working conditions, resulting in many samples with similar Doppler shifts. Table 4 shows that the recognition effects of Class I of the vessel and Class IV of the vessel are better than Class II of the vessel and Class III of the vessel, which may appear confusing.

The precision of Class I of the vessel is the highest, and the probability of incorrectly recognizing Class I of the vessel as Class I of the vessel is the highest because Class I of the vessel contains many straight samples and has a prominent stable spectrum without a Doppler shift. Class IV of the vessel has the highest recall, which indicates that the samples of different working conditions in Class IV are more balanced than the others and have more stable Doppler shift characteristics than the others.

#### 4.3.3. Visualization of Energy Distribution by the Architecture of camResNet

##### Power Spectral Density

**Figure 7:**
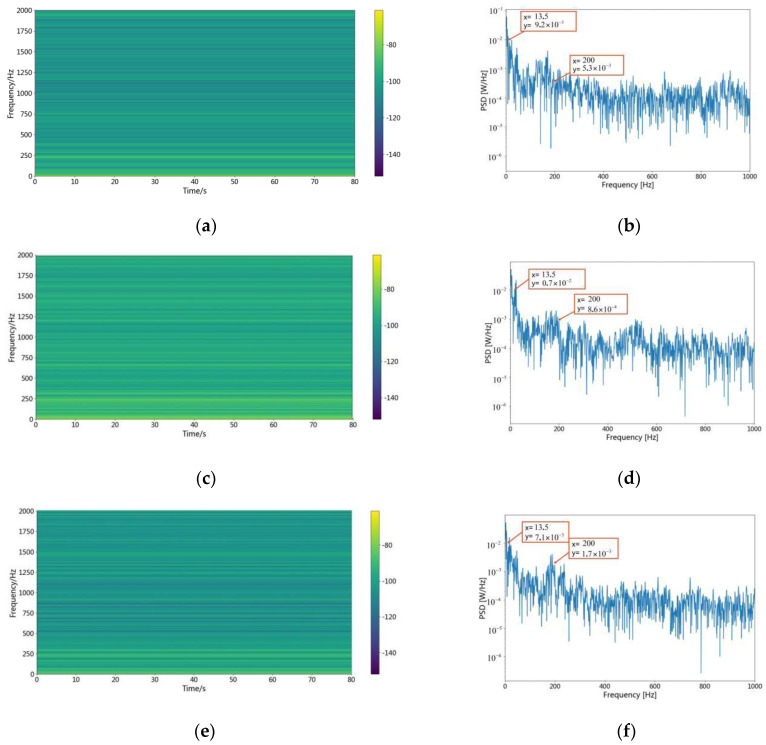
The spectrogram and power spectrum density of Class IV with camResNet. (**a**,**c**,**e**) The spectrogram of extracted features for Class IV vessel by camResNet; (**b**,**d**,**f**) The power spectrum density of extracted features for Class IV vessel by camResNet.

To further assess the feature extraction capability of the camResNet model, the trained camResNet model was fed by Class IV of the vessel because the spectrogram and the power spectral density are displayed in Figure 4 and Figure 5. Figure 7 shows the time-frequency diagram and the power spectral density of the output.

Figure 7a,c,e show the signal after the camResNet model by Class IV of the vessel, and Figure 4 shows the spectrogram of the original signal from Class IV vessel. The comparison indicates that the energy of the feature is still concentrated in the low frequency after the camResNet model. Figure 7b,d,f show the power spectrum density of Class IV of the vessel after processing the camResNet model, and Figure 5 shows the power spectrum density of the original signal for Class IV of the vessel. The comparison indicates that the apparent fundamental frequency signal in the original signal still exists after processing the camResNet model. In Figure 7, the camResNet model’s output contains not only stable signals but also some high-frequency signals, which indicates the camResNet model can avoid extracting unstable signals that are quickly Doppler shifted and recovers stable signals that are submerged in high frequencies.

##### t-SNE Feature Visualization Graphs

**Figure 8:**
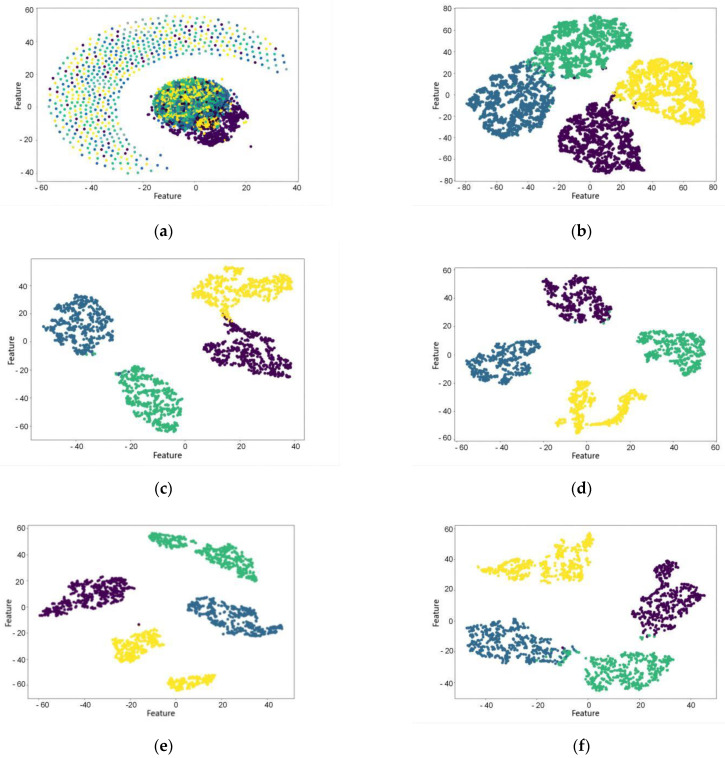
The t-SNE visualized graphs. (**a**) The t-SNE visualized graphs of original hydroacoustic signal; (**b**) The t-SNE visualized graphs’ output by camResNet; (**c**) The t-SNE visualized graphs of straight motion by camResNet; (**d**) The t-SNE visualized graphs of accelerating motion by camResNet; (**e**) The t-SNE visualized graphs of decelerating motion by camResNet; (**f**) The t-SNE visualized graphs of turning movement by camResNet.

The above experiment shows that the camResNet model can extract signals of stable frequencies in underwater acoustic signals. To further analyze the ability to extract features by camResNet, the distance of the original features and camResNet output features is visualized using the t-SNE method. Figure 8 shows the distance characteristics of the original signal and the output of the camResNet model when different working conditions are used as the input data. Figure 8a shows the t-SNE of the original underwater acoustic signal, which indicates that the original underwater acoustic signal has weak separability. Figure 8b shows the t-SNE of the output signals with the input of four different working conditions in the camResNet model. Figure 8c–f show the t-SNE of the output signals after putting straight motion, acceleration, deceleration, and turning conditions into the camResNet model, respectively.

From the figures, it can be seen that the camResNet model can classify all the working condition signals well and classify each kind of working condition signals well. In particular, the camResNet model can still classify the signals of acceleration conditions well when the acceleration conditions contain a large number of unstable Doppler shift signals. The classification maps show that the camResNet model can extract abstract and stable internal features under different conditions.

#### 4.3.4. Recognition Results for Different Data of camResNet

**Figure 9:**
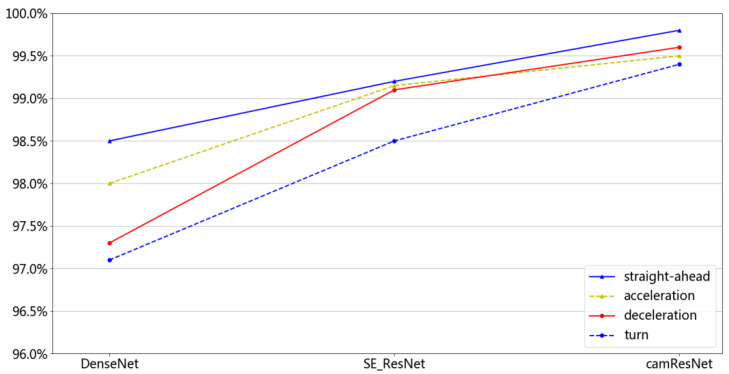
Recognition rate of cooperation targets.

Three different network models were used to compare the recognition results of underwater acoustic signals, which contained four working conditions. The models of DenseNet and SE_ResNet have a more extraordinary ability to recognize and were used for comparison with the camResNet model. The training method determines that the training and test data are the same—one of four working conditions. The recognition results were averaged by repeating the test five times, and the obtained experimental results are shown in Figure 9. The solid blue line is the recognition rate, which uses the data of straight motion working conditions as the training data and test data. The blue dotted line is the recognition rate, which uses the data of turn working conditions as the training data and test data. The solid red line is the recognition rate, which uses the data of deceleration working conditions as the training data and test data. The yellow dashed line is the recognition rate, which uses the data of acceleration working conditions as the training data and test data.

(1) The recognition rate of the camResNet model is higher than that of both the DenseNet model and the SE_ResNet model. The camResNet model can extract stable features that are effective for recognition.
(2) The recognition rate of the camResNet model under the straight motion condition is higher than under the other conditions, which indicates that the Doppler shift can affect the recognition of camResNet.
(3) There are different recognition rates with different working conditions containing different Doppler shifts. The maximum recognition rate of camResNet is 0,998; the minimum recognition rate is 0.994. The maximum recognition rate of DenseNet is 0.985, and the minimum recognition rate is 0.971. The decrease in recognition rate due to different Doppler shifts is smaller in the camResNet model than in the other models, which shows that the camResNet model has a better extraction of signals with Doppler shifts.

The network is trained and tested using data under one working condition, which is easy to overfit by a deeper model of DenseNet. The SE_ResNet model uses self-coding to compress channel features but does not consider the sparse characteristics of underwater acoustic targets. The camResNet model builds two different channel attention mechanisms, which fully consider the sparsity of underwater acoustic signal and the continuity spectrum, and they have better recognition results than the other models.

**Figure 10:**
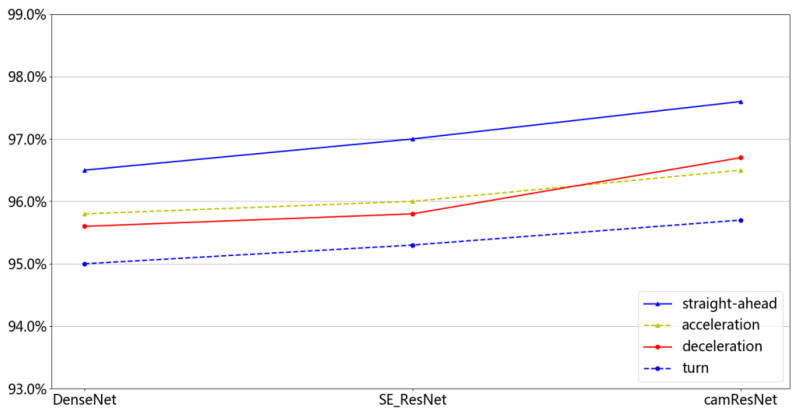
Recognition rate of non-cooperation targets.

The distributions of the training and test sets in the above experiments were identical. To further verify the recognition performance of the camResNet model, three network models were trained using four working conditions and tested under one working condition. The recognition results were averaged by repeating the test five times, and the obtained experimental results are shown in Figure 10. The solid blue line is the recognition rate, which uses the data of straight motion working conditions as the test data. The blue dotted line is the recognition rate, which uses the data of turn working conditions as the test data. The solid red line is the recognition rate, which uses the data of deceleration working conditions as the test data. The yellow dashed line is the recognition rate, which uses the data of acceleration working conditions as the test data.

(1) The maximum recognition rate of camResNet is 0,976; the minimum recognition rate is 0.965. The maximum recognition rate of DenseNet is 0.957, and the minimum recognition rate is 0.95. The recognition rate of the camResNet model is higher than that of the DenseNet model and the SE_ResNet model, and the performance is most evident under the deceleration condition.
(2) The recognition rates of the three network models vary smoothly under different working conditions, indicating that all three network models can extract stable signals from the initial signals and remove unstable frequency shifts. The camResNet model has the most robust ability from the recognition results.
(3) Compared with identical distributions of the training and test sets, the decrease in recognition rate due to different Doppler shifts becomes more prominent when the distributions of the training and test sets are not identical. This indicates that the recognition capabilities of the camResNet model with a Doppler shift are related to the distribution of training and test sets.

SE_ResNet uses compressed information to obtain channel weights to obtain certain stable features, so the recognition ability under different working conditions is better than that of DenseNet. The stable signal of the Doppler shift represents multi-scale information, which causes extract information with one scale to lose helpful information. The camResNet model uses convolution operation to extract channel information from two aspects. The first part uses the convolution kernel superposition to expand the perceptual field and extract features of different scales. The second part extracts the feature from the local features of all information. Fusing the two features as the weights of channels can comprehensively extract the stable features under the Doppler frequency shift. Hence, the camResNet model has better recognition results for different working conditions data containing the Doppler frequency shift information.

## 5. Conclusions

The camResNet model adds a channel attention mechanism to the ResNet model based on the characteristics of underwater acoustic signals. This channel attention mechanism can enhance the stable spectral features and remove the unstable signals caused by the Doppler shifts. The experiments compare the recognition ability of six different deep-learning models under different Doppler shift frequencies. The results show that the recognition rate of the camResNet model is higher than that of the other network models. The camResNet model has a recognition rate of 98.2%, which is 1.1–15.8% higher than the other networks. The precision, recall rate, F1-score, and accuracy are used to demonstrate that the data used in the experiments are balanced between the classes and that the experimental results are valid. Test the effectiveness of the proposed method with the same distribution and different distributions for the training and test sets. The three network models with better recognition results are selected for testing. In the same training set and test set distribution, the recognition rate of camResNet varies from 0.003 to 0.023 for different working conditions. In contrast, the recognition rate of DenseNet varies from 0.015 to 0.019 for different distributions of the training set and test set. The results show that the proposed method is more suitable when the training and test sets are identically distributed. Further, using visualization methods to learn the features of the signal extracted by the camResNet model, the results show that the camResNet model can extract the stable multi-group harmonic signals and restore some weak high-frequency stable signals in the original signal.

The camResNet model can effectively extract the features of underwater acoustic signals with the Doppler shift. The following work will use the camResNet model to recognize the underwater acoustic signals with the Doppler shift for small samples, solving the problem of data-driven underwater acoustic signals in deep learning.

## Figures and Tables

**Table 1:** Recognition rate for different numbers of residual layers.

Residual Layers.	1	2	3	4
Recognition rate of test set	96.3%	98.2%	96.5%	91.4%

**Table 2:** Recognition rate for convolution kernels of different sizes.

Convolution Kernels	Recognition Rate of Validated Set	Recognition Rate of Test Set
1×3	91.6%	91.1%
1×5	92.0%	91.5%
1×7	94.3%	92.1%
1×11	97.1%	92.3%
1×15	98.2%	93.1%
1×17	97.9%	93.5%
1×21	97.8%	95.3%
1×25	98.1%	95.3%
1×33	98.1%	95.9%
1×41	98.2%	96.1%
1×49	98.3%	96.3%
1×57	99.5%	97.4%
1×64	99.9%	98.2%
1×75	98.9%	97.1%
1×85	98.3%	96.4%
1×95	99.5%	95.9%

**Table 3:** Recognition rate of proposed model and compared models with straight motion data and four different working conditions.

The Input	Models	Recognition Rate	
		Straight Motion Data	Four Different Working Conditions
Time domain signal	camResNet	98.9%	98.2%
Frequency domain signal	DBN	85.6%	82.4%
Time domain signal	GAN	96.6%	96.3%
Frequency domain signal	DenseNet	97.3%	96.1%
Time domain signal	U_Net	93.9%	93.6%
Time domain signal	SE_ResNet	98.8%	97.1%

**Table 4:** Recognition results of camResNet.

	Precision	Recall	F1-Score	Accuracy
Class I vessel	0.996	0.991	0.993	0.996
Class II vessel	0.984	0.981	0.982	0.991
Class III vessel	0.978	0.982	0.980	0.990
Class IV vessel	0.993	0.998	0.995	0.997

**Table 5:** Confusion matrix of camResNet.

	Class I Vessel	Class II Vessel	Class III Vessel	Class IV Vessel
Class I vessel	1783	1	7	9
Class II vessel	2	1764	32	2
Class III vessel	3	24	1772	1
Class IV vessel	2	0	1	1769

## Data Availability

Not applicable.
